# Perceptions of Organizational Affect and Holistic Mental Health in United States Army Soldiers During and After Deployment

**DOI:** 10.1002/smi.70097

**Published:** 2025-08-04

**Authors:** Walter J. Sowden, Rachell L. Jones, Stein P. Thorbeck

**Affiliations:** ^1^ Ross School of Business University of Michigan Ann Arbor Michigan USA; ^2^ Walter Reed Army Institute of Research Silver Spring Maryland USA; ^3^ 82nd Airborne Division U.S. Army Fort Bragg North Carolina USA

**Keywords:** deployment, holistic mental health, military, organizational affect, social perception

## Abstract

This study examined how U.S. Army Soldiers' subjective perceptions of organizational affect relate to holistic mental health across the deployment cycle. Using a repeated cross‐sectional design, 1554 Soldiers completed surveys before (T1), during (T2), and after (T3) deployment. Perceptions of organizational affect were conceptualised along two theoretically grounded dimensions: *ambiance* (emotional tone) and *vigour* (motivational energy), and measured using a novel validated scale. Factor analyses identified four distinct perceptions: *suffering* (negative ambiance, low vigour), *contentment* (positive ambiance, low vigour), *restlessness* (negative ambiance, high vigour), and *zeal* (positive ambiance, high vigour). General linear models showed that perceptions of organizational affect at T2 and T3 were significantly associated with psychological distress, hedonic happiness, and eudaimonic thriving at those time points, controlling for pre‐deployment (T1) holistic mental health. Perceptions of suffering were linked to increased distress, perceptions of contentment to greater happiness, and perceptions of both zeal and restlessness to greater thriving. Perceptions of organizational affect accounted for 5%–15% of the variance in holistic mental health outcomes. These findings underscore the importance of both perceptions of organizational ambiance and vigour in shaping mental health and well‐being and suggest opportunities for targeted interventions in high‐stakes occupational environments like military deployments.

## 1 Introduction

High‐stakes occupational environments, such as military deployments, place extraordinary demands on individuals, often leading to significant mental health challenges (Pietrzak et al. 2012). Service members experience higher rates of *psychological distress*, including depression, anxiety, and post‐traumatic stress disorder, compared to civilian populations (Inoue et al. 2023). However, mental health in these contexts extends beyond distress to include positive states of well‐being, such as *hedonic happiness*, which involves experiencing positive emotions and life satisfaction (Kahneman et al. 1999), and eudaimonic thriving, which involves pursuing meaning, purpose, and self‐actualisation (Ryan and Deci 2001). Understanding the factors that contribute to *holistic mental health*, encompassing both the alleviation of distress and the promotion of well‐being (Jahoda 1958), is crucial for supporting individuals and enhancing organizational effectiveness in high‐stakes environments, such as military deployments (Gutierrez and Adler 2022).

One critical but understudied factor influencing holistic mental health is *perceptions of organizational affect*: the emotional gestalt of an organisation as subjectively experienced by its members. Unlike organizational culture or climate, which emphasise values or norms, organizational affect reflects the collective emotional tone within the organisation (S. G. Barsade and Knight 2015; Collins et al. 2013; Elfenbein 2023). In high‐stakes settings, where emotional contagion and collective morale significantly influence performance and resilience (Britt and Dickinson 2005; Tee 2015), service members' perceptions of their unit's affective environment can substantially impact their psychological health and performance (Adler and Sowden 2018; Sowden et al. 2022).

Despite extensive research on resilience, leadership, and cohesion in military contexts (Brooks and Greenberg 2018; Campbell‐Sills et al. 2022), there is a lack of understanding about how service members perceive the collective emotional tone of their organisation and how these perceptions relate to holistic mental health outcomes. This study addresses these gaps by examining perceptions of organizational affect and their relationship with psychological distress, hedonic happiness, and eudaimonic thriving across the military deployment cycle, a process that includes operational stressors during deployment and the psychological challenges of reintegration after returning home, both of which can meaningfully influence service members' holistic mental health (Adler and Castro 2013; Gutierrez and Adler 2022).

### 1.1 Theoretical Foundations and Research Gaps

Service members' subjective interpretations of their organizational environment play a critical role in shaping their psychological health and resilience (Adler and Castro 2013; Ganz et al. 2021). Although organizational research has extensively examined culture, climate, and leadership (S. Barsade and O’Neill 2016; Schneider and Barbera 2014; Schein 2010), few studies have explored how individuals perceive the emotional qualities of their organisation and how such perceptions influence both psychological distress and positive well‐being. In civilian settings, research suggests that affective workplace climates impact job satisfaction, engagement, and mental health (James et al. 2021; Maslach and Leiter 2016; Men and Robinson 2018). However, comparable research in military settings is scarce (for an exception, see Adler et al. 2022). Given the unique stressors of military deployments, including social isolation, life‐or‐death decision‐making, and operational stress, the relationship between organizational affect and well‐being may operate differently in military contexts compared to civilian workplaces.

To address these gaps, we investigate two primary research questions: (1) What is the underlying psychological dimensionality of perceptions of organizational affect, and (2) How do these perceptions relate to psychological distress, hedonic happiness, and eudaimonic thriving during and after a military deployment? Although we examine during‐ and post‐deployment phases separately to explore whether these relationships varied across the deployment cycle, we do not propose formal hypotheses about differences between time points.

### 1.2 The Two‐Dimensional Structure of Perceptions of Organizational Affect

Building on Social Perception Theory (Fiske et al. 2007) and Core Affect Theory (Russell 2003), we propose that perceptions of organizational affect emerge along two fundamental dimensions. Social Perception Theory suggests that individuals evaluate social entities, including organisations, based on *warmth* (perceived intent) and *competence* (perceived capability). We align warmth with *emotional ambiance* (positive vs. negative tone) and competence with *motivational vigour* (high vs. low energy). This alignment is supported by research showing that perceptions of competence often reflect not just skill or ability, but also the perceived energy, initiative, and efficacy with which goals are pursued, which are core features of motivational vigour (Cuddy et al. 2011; Wojciszke et al. 1998). Similarly, Core Affect Theory conceptualises emotions along dimensions of *valence* (pleasant‐unpleasant) and *activation* (high‐low energy), mirroring the ambiance and vigour structure.

Thus, ambiance reflects the perceived emotional tone of the organisation (Collins et al. 2013), ranging from positive (e.g., congenial, supportive) to negative (e.g., disgruntled, hostile), while vigour reflects the perceived motivational energy of the organisation (Shirom 2011), ranging from high (e.g., exuberant, proactive) to low (e.g., lethargic, passive).

Based on this framework, we hypothesise that perceptions of organizational affect exhibit a two‐dimensional structure reflecting ambiance (valence) and vigour (activation) (Hypothesis 1). If this structure is supported, we further expect that combinations of these two dimensions give rise to four distinct patterns of perceived organizational affect (Hypothesis 2): *Suffering* (negative ambiance, low vigour), *Contentment* (positive ambiance, low vigour), *Zeal* (positive ambiance, high vigour), and *Restlessness* (negative ambiance, high vigour). These four perceptions can be organised into a 2 × 2 conceptual framework crossing ambiance (positive vs. negative) with vigour (high vs. low), as shown in Table 1. Although Hypotheses 1 and 2 are conceptually distinct, they are empirically connected: Hypothesis 1 focuses on the underlying dimensional structure of organizational affect, while Hypothesis 2 interprets how combinations of those dimensions give rise to theoretically meaningful patterns. These expectations are tested in tandem using a combination of factor analysis and theoretical modelling.

**TABLE 1:** Perceptions of organizational affect by ambiance and vigour.

	Positive ambiance	Negative ambiance
High vigour	*Zeal* Supportive, engaged environment. Likely to reduce distress and promote both happiness and thriving.	*Restlessness* Energised but tense environment. May increase distress; can support thriving under challenging conditions.
Low vigour	*Contentment* Pleasant but passive environment. Likely to reduce distress and promote happiness; may limit thriving.	*Suffering* Demoralised, depleted environment. Likely to increase distress, reduce happiness, and hinder thriving.

*Note:* The model organizes perceptions of organizational affect into four patterns based on two dimensions: ambiance (positive vs. negative emotional tone) and vigour (high vs. low motivational energy). These combinations yield four profiles: suffering, restlessness, contentment, and zeal, each with distinct implications for psychological distress, hedonic happiness, and eudaimonic thriving.

### 1.3 Perceptions of Organizational Affect and Holistic Mental Health

Why do these perceptions matter for holistic mental health? Extensive research suggests that emotion and motivation are deeply intertwined, dynamically shaping well‐being and performance (Ryan and Deci 2000). Emotions fuel motivation by signalling needs, directing attention, and energising action, while motivation regulates emotional responses through goal‐directed behaviour (Carver and Scheier 1990).

Positive ambiance fosters happiness and reduces distress, creating a psychologically supportive environment where individuals experience greater emotional stability and social cohesion (S. G. Barsade and O’Neill 2014; Fredrickson 1998). Conversely, negative ambiance exacerbates distress, undermines morale, and weakens interpersonal connections (Ashkanasy and Härtel 2014; Men and Robinson 2018), making it more difficult for individuals to cope with stressors in the workplace (Lazarus and Folkman 1984; Maslach and Leiter 2016). High vigour promotes engagement, problem‐solving, and personal growth by enabling individuals to persist through challenges and derive meaning from their work, processes that, in turn, generate positive emotions and reinforce motivation and resilience over time (Adrian et al. 2018; Shirom 2011). In contrast, low vigour leads to disengagement and passivity, contributing to stagnation and a decline in well‐being (Maslach and Leiter 2016).

While positive ambiance is generally associated with reduced distress and increased happiness, and high vigour is linked to greater engagement and thriving, we predict that their interaction will yield unique psychological effects hypothesised to distinctly influence aspects of holistic mental health. Drawing on emotion‐motivation theories (Ryan and Deci 2000; Fredrickson 1998; LePine et al. 2005; Lazarus and Folkman 1984; Carver and Scheier 1990), we expect the following:

Perceptions of organizational suffering (negative ambiance, low vigour) reflect an emotionally negative and motivationally depleted environment. Cognitive Appraisal Theory (Lazarus and Folkman 1984) suggests that such environments are likely to be perceived as threatening or demoralising, increasing psychological distress. The absence of positive ambiance further reduces opportunities for hedonic happiness, while low vigour diminishes motivation, undermining eudaimonic thriving.

Perceptions of organizational contentment (positive ambiance, low vigour) capture a pleasant but subdued organizational climate. Broaden‐and‐Build Theory (Fredrickson 1998) suggests that positive ambiance fosters hedonic happiness and reduces distress. However, low vigour limits individuals' motivation for pursuing purpose or meaning, weakening their relationship with eudaimonic thriving (Huta and Ryan 2010).

Perceptions of organizational zeal (positive ambiance, high vigour) represent an emotionally positive and highly energetic environment. Self‐Determination Theory (Ryan and Deci 2000) suggests that high vigour fuels engagement and motivation, while positive ambiance reduces distress and enhances happiness (Aboramadan and Kundi 2023). In such environments, eudaimonic thriving is strengthened as individuals experience greater autonomy, competence, and purpose‐driven engagement.

Perceptions of organizational restlessness (negative ambiance, high vigour) reflect a tense yet energised organizational climate. While negative ambiance is typically associated with distress and reduced happiness (Collins et al. 2013), high vigour may motivate individuals to overcome adversity, driving eudaimonic thriving despite emotional strain. This view aligns with Challenge‐Hindrance Stressor Theory (Cavanaugh et al. 2000; LePine et al. 2005) and Eustress Theory (Selye 1974), which suggest that certain negatively valenced affective climates, when paired with high vigour, may foster resilience, adaptability, and growth under the right conditions.

Based on the theoretical reasoning above, we hypothesise the following associations between perceptions of organizational affect and holistic mental health outcomes. Each hypothesis reflects the expected relationships between a specific affective perception and the three outcome domains of psychological distress, hedonic happiness, and eudaimonic thriving.

Hypothesis 3a (Suffering): Perceptions of organizational suffering will be positively associated with psychological distress and negatively associated with hedonic happiness and eudaimonic thriving.

Hypothesis 3b (Contentment): Perceptions of organizational contentment will be positively associated with hedonic happiness and will show weak or non‐significant associations with psychological distress and eudaimonic thriving.

Hypothesis 3c (Zeal): Perceptions of organizational zeal will be negatively associated with psychological distress and positively associated with hedonic happiness and eudaimonic thriving.

Hypothesis 3d (Restlessness): Perceptions of organizational restlessness will be positively associated with eudaimonic thriving and show weak or non‐significant associations with psychological distress and hedonic happiness.

While these hypotheses are grounded in established theories of emotion, motivation, and occupational stress, we acknowledge that this examination is partly exploratory in nature. The proposed 2 × 2 framework and the resulting affective patterns have not been empirically examined in military contexts. Therefore, Hypotheses 3a–3d should be interpreted as theoretically informed with exploratory predictions, reflecting our aim to generate insight into how perceptions of organizational affect may relate to holistic mental health outcomes in high‐stakes environments. By integrating emotion‐motivation theories with research on organizational affect, this study presents a novel framework for understanding how perceptions of organizational affect influence service members' holistic mental health throughout the deployment cycle.

## 2 Method

### 2.1 Participants and Procedure

Using a repeated cross‐sectional design, this study examined how U.S. Army Soldiers' subjective perceptions of organizational affect related to their holistic mental health across the deployment cycle. Participants were convenience samples of U.S. Army Soldiers from an Armoured Brigade Combat Team (ABCT) who completed a survey at one or more of the three time points: pre‐deployment (T1, two months before deployment), mid‐deployment (T2, 4 months into deployment), and post‐deployment (T3, 1 month after returning to the U.S.). Due to operational constraints, different subsets of soldiers were sampled at each time point, and individuals were not tracked across waves. Rather than modelling organizational‐level phenomena, this study focused on Soldiers' perceptions of their affective environment within their organisation and how these perceptions are related to their holistic mental health outcomes. Analyses examined the relationship between perceptions of organizational affect and psychological distress, hedonic happiness, and eudaimonic thriving at T2 and T3, while controlling for individual differences in pre‐deployment (T1) holistic mental health. This design leveraged the naturally unfolding context of a military deployment to assess how subjective experiences of organizational affect relate to holistic mental health outcomes across different stages of deployment.

1554 soldiers consented to the research and completed the T1 survey. Of these, 488 soldiers (31.4%) completed the T2 survey, and 408 (26.3%) completed the T3 survey. Participants were recruited through unit informational briefings led by research staff, with survey completion taking place during scheduled training sessions. Participation was voluntary, and no incentives were provided. The study adhered to the ethical guidelines for research involving human participants and was approved by an accredited institutional review board. Informed consent was obtained from all participants. Descriptive statistics for key demographic characteristics, psychological health variables, and perceptions of organizational affect at each time point are presented in Table 2. These values reflect unstandardised scale scores, enabling meaningful and practical interpretation. To aid transparency and interpretation, we also provide a summary of differences in sample composition across time points, enabling readers to assess the potential impact of sample attrition on study findings.

**TABLE 2:** Descriptive statistics for sample characteristics and study variables at pre‐deployment (T1), mid‐deployment (T2), and post‐deployment (T3).

Variable	T1 (*n* = 1554)	T2 (*n* = 488)	T3 (*n* = 408)
Age (years)	25.02 (5.92)	25.09 (5.68)	25.13 (5.82)
Gender (% male)	91%	92.10%	92.90%
Race (% white)	49.10%	49.90%	52.20%
Rank (% junior enlisted)	62.20%	66.50%	66.90%
Psychological distress	10.68 (4.23)	11.10 (4.50)	10.74 (4.43)
Hedonic happiness	3.67 (0.89)	3.58 (0.90)	3.62 (0.92)
Eudaimonic thriving	4.25 (0.73)	4.14 (0.75)	4.09 (0.73)
Organizational suffering	2.83 (0.85)	2.89 (0.86)	2.90 (0.83)
Organizational contentment	2.59 (0.83)	2.60 (0.80)	2.60 (0.80)
Organizational zeal	2.90 (0.75)	2.95 (0.70)	2.96 (0.72)
Organizational restlessness	3.84 (0.81)	3.86 (0.79)	3.93 (0.76)

*Note:* Values represent means and standard deviations (in parentheses), except for demographic percentages. Mental health and organizational affect variables are reported using raw (unstandardised) scale values.

To assess whether attrition across the deployment cycle introduced bias, we compared participants who completed the T2 and T3 surveys with those who did not on key demographic and baseline measures. No statistically significant differences were found in age, gender, race, rank, or baseline levels of psychological distress, hedonic happiness, or eudaimonic thriving (all *p'*s > 0.05). However, participants who completed the post‐deployment survey reported slightly lower perceptions of organizational suffering at T1 compared to those who did not, *t* (1114) = 2.86, *p* = 0.004. All other differences in perceptions of organizational affect were non‐significant. These results suggest that attrition was largely random, with minimal evidence of systematic dropout bias.

### 2.2 Measures

*Psychological Distress* was assessed using a composite index of anxiety, depression, and PTSD symptoms, reflecting the general psychopathology factor (‘p factor’; Caspi et al. 2014). Scores from the Generalised Anxiety Disorder Scale (GAD‐7; Spitzer et al. 2006; *α* = 0.92–0.93), the Patient Health Questionnaire (PHQ‐9; Kroenke and Spritzer 2002; *α* = 0.90–0.91), and the PTSD Checklist ‐ Military Version (PCL‐MS; Bliese et al. 2008; *α* = 0.88–0.91), were each standardized (z‐scored) and then averaged to form the composite. This approach ensured that each component contributed equally, preventing scales with larger ranges (e.g., the PCL‐MS) from disproportionately influencing the composite index. The resulting psychological distress variable was used in standardized form in all analyses. Higher composite scores indicated greater psychological distress.

*Eudaimonic Thriving* was measured using the 42‐item Psychological Well‐Being Scale (Ryff and Keyes 1995; *α* = 0.94), which assesses autonomy, mastery, personal growth, relationships, self‐acceptance, and purpose in life. Scores were averaged to create a total scale score (range: 1–6), with higher scores indicating greater thriving. This total score was used in raw form in all analyses. Prior research supports the unidimensional use of this measure in military samples (Trachik et al. 2023).

*Hedonic Happiness* was assessed using a composite index combining the Satisfaction with Life Scale (SWLS; Diener et al. 1985; *α* = 0.91) and the Positive and Negative Affect Schedule ‐ Short Form (PANAS‐SF; Thompson 2007; αPA = 0.72; αNA = 0.75). Scores from the SWLS and the positive affect subscale of the PANAS were standardized (z‐scores) and averaged with the reverse‐scored and standardized negative affect subscale to create a composite reflecting overall hedonic well‐being. This standardized composite score was used in all analyses. Prior research has validated this composite approach as a reliable operationalisation of subjective well‐being (Busseri and Sadava 2011).

*Perceptions of Organizational Affect* were measured using a modified 48‐item version of the Emotional Cultures Scale (Barsade and O'Neill 2014), adapted for military contexts (Adler et al. 2022; *α* = 0.91). Soldiers responded to the prompt, ‘To what degree do the Soldiers in your brigade express the following emotions at work?’ and rated the perceived frequency of 48 specific emotions expressed on a 5‐point Likert scale (1 = never to 5 = very often). Items assessed a wide range of positive and negative emotions to capture how Soldiers perceived the emotional tone and energy of the organizational environment. As noted in the introduction, perceptions of organizational affect are inherently subjective, reflecting individuals' interpretations of their collective environment rather than an objective assessment.

*Covariates.* Demographic variables (age, gender, race, and rank) were collected at T1. A measure of social desirability (Reynolds 1982) was included at T2 (*α* = 0.73) and T3 (*α* = 0.77) to account for potential response bias. A participant attention check was administered at T1 to promote data quality (Meade and Craig 2012).

### 2.3 Analytic Strategy

Analyses were conducted in SPSS (Version 28) and R (Version 4.4.0, ‘lavaan’ package; Rosseel 2012). Exploratory Factor Analysis (EFA) was used on the T1 data to examine the dimensionality of perceptions of organizational affect, followed by confirmatory factor analyses (CFA) on the T2 and T3 data to validate the structure. To assess the potential impact of attrition on the factor structure, a follow‐up exploratory factor analysis (EFA) was conducted on a restricted T1 subsample, which included only those participants who also provided data at T2 or T3. This analysis produced the same four‐factor solution as the full T1 sample, suggesting that dropout patterns did not meaningfully affect the factor structure.

General linear models (GLMs) tested how perceptions of organizational affect at T2 and T3 were associated with contemporaneous levels of psychological distress, hedonic happiness, and eudaimonic thriving. To account for pre‐deployment individual differences, T1 mental health scores were included as covariates. This adjustment enabled a more rigorous assessment of whether perceptions of organizational affect predicted concurrent mental health outcomes during and after deployment, above and beyond baseline well‐being. This design leveraged the naturally unfolding deployment cycle while acknowledging the repeated cross‐sectional nature of the data.

Psychological distress and hedonic happiness composites were standardized prior to analysis, while eudaimonic thriving and perceptions of organizational affect were analysed in their raw scale forms. T2 and T3 outcomes were analysed separately as a robustness check to assess whether associations between organizational affect and well‐being were consistent across the operational (T2) and reintegration (T3) phases of the deployment cycle. Missing data was minimal (< 5% across variables) and handled using mean imputation, consistent with best practices for data assumed to be missing at random (Tabachnick and Fidell 2018). Sensitivity analyses excluding imputed values and covariates confirmed the robustness of the findings.

## 3 Results

### 3.1 Factor Structure of Perceptions of Organizational Affect

An EFA was conducted using Principal Axis Factoring with an Oblimin rotation on the T1 dataset. Parallel analysis and scree plot inspection supported a four‐factor solution, χ^2^ (855) = 3,214, *p* < 0.001, which explained 42.1% of the variance. The factor analysis identified four distinct factors: (1) emotional withdrawal, (2) motivational energy, (3) emotional satisfaction, and (4) frustration‐related affect. Items with low factor loadings (< 0.40) or significant cross‐loadings (< 0.30) were excluded for clarity. See Table 3 for item loadings.

**TABLE 3:** Factor loadings from the exploratory factor analysis of the Emotional Cultures Scale measured before deployment.

Item	Withdrawal	Energy	Satisfaction	Frustration
Being scared	0.763			
Loneliness	0.690			
Isolation	0.632			
Nervousness	0.631			
Grief	0.614			
Depression	0.612			
Shame	0.597			
Guilt	0.583			
Fear	0.514			
Jealousy	0.461			
Gloominess	0.452			0.341
Hostility	0.444			
Remorse	0.427			
Sadness	0.401			0.315
Alertness		0.721		
Attentiveness		0.673		
Triumph		0.654		
Pride		0.653		
Determination		0.648		
Being active		0.595		
Being inspired		0.547		
Good humour		0.541		
Optimism		0.531		
Eagerness		0.517		
Hope		0.467		
Amusement		0.431		
Compassion		0.376		
Joy			0.820	
Cheerfulness			0.783	
Happiness			0.773	
Enthusiasm			0.718	
Excitement			0.711	
Caring			0.349	
Frustration				0.800
Annoyance				0.769
Anger				0.644
Grumpiness				0.571
Irritation				0.437
Being upset	0.380			0.454
Anxiety	0.336			0.336
Arrogance				0.339
Snobbishness				
Unfriendliness				
Envy				
Tenderness				
Humility				
Light‐hearted				
Affection				

*Note:* EFA conducted on T1 data (*n* = 1554). Principal Axis Factoring extraction with Oblimin rotation was used. Factor labels reflect the primary emotional and motivational dimensions that were identified. Loadings below 0.3 are not shown.

A CFA on the T2 and T3 datasets validated the factor structure using an optimised four‐factor model with five items per factor. Model fit was acceptable at both time points: T2, χ^2^ (164) = 1070, CFI = 0.933, TLI = 0.923, SRMR = 0.0542, RMSEA = 0.0618, and T3, χ^2^ (164) = 1601, CFI = 0.924, TLI = 0.912, SRMR = 0.0599, RMSEA = 0.0693. Sensitivity analyses confirmed that the factor structure observed in the full T1 sample was replicated among the subset of participants who completed at least one follow‐up wave. Factor loadings and structure were consistent, indicating that attrition did not meaningfully distort the factor solution.

### 3.2 Ambiance and Vigour: Conceptual Alignment

**FIGURE 1:**
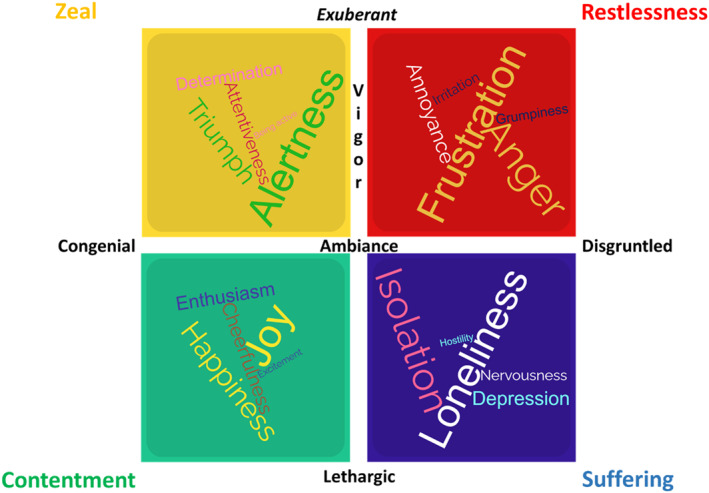
Illustrative word clouds representing perceptions of organizational affect by ambiance (congenial; disgruntled) and vigour (exuberant; lethargic). This figure illustrates the four perceptions of organizational affect: zeal, contentment, restlessness, and suffering, based on a two‐dimensional structure of ambiance and vigour. Words in each cloud represent Emotional Cultures Scale items with the highest factor loadings, minimal cross‐loadings, and strongest model fit, as determined by exploratory and confirmatory factor analyses. Portions of this figure were generated using WordClouds.com.

The factor structure aligned with two theoretical dimensions. Factors 1 and 4 (withdrawal and frustration) reflected negative ambiance (disgruntled), whereas Factors 2 and 3 (energy and satisfaction) represent a positive ambiance (congenial). Simultaneously, Factor 1 (withdrawal) and Factor 3 (satisfaction) reflect low vigour (lethargic), while Factor 2 (energy) and Factor 4 (frustration) reflect high vigour (exuberant). This two‐dimensional structure supports H1. The resulting perceptions: zeal (congenial, exuberant), contentment (congenial, lethargic), restlessness (disgruntled, exuberant), and suffering (disgruntled, lethargic), are consistent with prior models of emotional culture and social evaluation and support H2 (see Figure 1).

### 3.3 Associations Between Perceptions of Organizational Affect and Holistic Mental Health

General linear models (GLMs) were used to assess whether perceptions of organizational affect predicted holistic mental health outcomes at T2 and T3. Predictors included the four factor scores; covariates included T1 mental health scores and demographics. Mid‐ and post‐deployment outcomes were analysed separately to assess whether the associations between perceptions of organizational affect and holistic mental health remained stable across different stages of the deployment cycle. Perceptions of organizational affect accounted for significant variance in holistic mental health outcomes at T2: psychological distress, 9.3%; eudaimonic thriving, 5.5%; hedonic happiness, 5.2%; and at T3: psychological distress, 6.4%; eudaimonic thriving, 5.3%; hedonic happiness, 14.7%. See Table 4 for unstandardised coefficients and significance levels.

**TABLE 4:** The relationship between perceived organizational affect, categorised into suffering, contentment, zeal, and restlessness, and the three dimensions of holistic mental health during and after deployment.

	Psychological distress	Eudaimonic thriving	Hedonic happiness
During deployment			
Suffering	*B* = 2.77, *p* = 0.009	*B* = −0.141, *p* = 0.007	*B* = −0.048, *p* = 0.452
Contentment	*B* = −1.37, *p* = 0.226	*B* = −0.026, *p* = 0.641	*B* = 1.54, *p* = 0.047
Zeal	*B* = −3.09, *p* = 0.010	*B* = 1.65, *p* = 0.006	*B* = 1.05, *p* = 0.164
Restlessness	*B* = 1.29, *p* = 0.267	*B* = 0.168, *p* = 0.004	*B* = −0.072, *p* = 0.307
Explained variance	9.3%	5.5%	5.2%
After deployment			
Suffering	*B* = 4.10, *p* < 0.001	*B* = −0.122, *p* = 0.029	*B* = −0.114, *p* = 0.048
Contentment	*B* = −1.06, *p* = 0.294	*B* = 0.051, *p* = 0.392	*B* = 0.225, *p* < 0.001
Zeal	*B* = −0.249, *p* = 0.831	*B* = 0.147, *p* = 0.032	*B* = 0.060, *p* = 0.383
Restlessness	*B* = −0.122, *p* = 0.213	*B* = 0.164, *p* = 0.005	*B* = −0.142, *p* = 0.028
Explained variance	6.4%	5.3%	14.7%

*Note:* All models controlled for baseline (T1) levels of the respective outcome variable (psychological distress, eudaimonic thriving, or hedonic happiness), as well as participant age, gender, military rank, and social desirability. *B* = unstandardised regression coefficient; *p* = significance level.

Organizational suffering significantly predicted increased psychological distress and decreased eudaimonic thriving at both time points, consistent with H3a. Suffering did not significantly predict hedonic happiness during deployment, suggesting that distress increased without a corresponding decline in life satisfaction. Contentment was positively associated with hedonic happiness but was not significantly related to distress or thriving at both time points, partially supporting H3b. Zeal was consistently and positively associated with eudaimonic thriving but not with distress or happiness, partially supporting H3c. Similarly, H3d was partially supported, as organizational restlessness was positively associated with eudaimonic thriving at both time points. However, it was not significantly associated with distress at either time point or happiness during deployment.

### 3.4 Robustness Checks

Sensitivity analyses were conducted by (1) removing covariates, (2) removing the attention check filter, or (3) using raw (non‐imputed) data. Results were largely consistent across all models. Refer to Table 5 for detailed results across various robustness conditions.

**TABLE 5:** Robustness of associations between perceived organizational affect and mental health outcomes under alternative analytic specifications.

	Covariates excluded	Attention check removed	Raw (vs. imputed) data
Mid‐deployment psychological distress			
Suffering	*B* = 3.28, *p* < 0.001	*B* = 2.91, *p* = 0.003	*B* = 3.061, *p* = 0.005
Zeal	*B* = −2.94, *p* = 0.006	*B* = −2.75, *p* = 0.010	*B* = −2.88, *p* = 0.018
Contentment	*B* = −1.08, *p* = 0.295	*B* = −1.11, *p* = 0.288	*B* = −1.48, *p* = 0.203
Restlessness	*B* = 0.605, *p* = 0.565	*B* = 1.68, *p* = 0.110	*B* = 0.833, *p* = 0.488
Explained variance	9.4%	9.6%	8.8%
Mid‐deployment eudaimonic thriving			
Suffering	*B* = −2.20, *p* < 0.001	*B* = −0.148, *p* = 0.001	*B* = −0.154, *p* = 0.004
Zeal	*B* = 0.109, *p* = 0.043	*B* = −0.148, *p* = 0.002	*B* = 0.140, *p* = 0.026
Contentment	*B* = 0.019, *p* = 0.714	*B* = −0.044, *p* = 0.382	*B* = −0.028, *p* = 0.642
Restlessness	*B* = 0.207, *p* < 0.001	*B* = 0.171, *p* < 0.001	*B* = 0.160, *p* = 0.010
Explained variance	6.0%	4.6%	3.6%
Mid‐deployment hedonic happiness			
Suffering	*B* = −0.172, *p* = 0.002	*B* = −0.041, *p* = 0.436	*B* = −0.065, *p* = 0.323
Zeal	*B* = 0.214, *p* = 0.001	*B* = 0.102, *p* = 0.092	*B* = 0.050, *p* = 0.520
Contentment	*B* = 0.022, *p* = 0.748	*B* = 0.204, *p* < 0.001	*B* = 0.174, *p* = 0.030
Restlessness	*B* = −0.059, *p* = 0.359	*B* = −0.073, *p* = 0.192	*B* = 0.016, *p* = 0.843
Explained variance	8.4%	7.3%	3.4%
Post‐deployment psychological distress			
Suffering	*B* = 4.85, *p* < 0.001	*B* = 4.77, *p* < 0.001	*B* = 3.98, *p* < 0.001
Zeal	*B* = −1.38, *p* = 0.192	*B* = 0.444, *p* = 0.673	*B* = −0.595, *p* = 0.624
Contentment	*B* = −0.195, *p* = 0.835	*B* = −1.49, *p* = 0.123	*B* = −0.886, *p* = 0.400
Restlessness	*B* = −0.947, *p* = 0.319	*B* = −1.00, *p* = 0.302	*B* = –1.16, *p* = 0.256
Explained variance	10.4%	8.1%	6.2%
Post‐deployment eudaimonic thriving			
Suffering	*B* = −0.156, *p* = 0.003	*B* = −0.111, *p* = 0.025	*B* = –0.095, *p* = 0.117
Zeal	*B* = 0.114, *p* = 0.063	*B* = −0.069, *p* = 0.026	*B* = 0.160, *p* = 0.034
Contentment	*B* = 0.076, *p* = 0.164	*B* = 0.057, *p* = 0.470	*B* = 0.045, *p* = 0.500
Restlessness	*B* = 0.134, *p* = 0.015	*B* = 0.053, *p* = 0.005	*B* = 0.142, *p* = 0.019
Explained variance	6.1%	4.3%	4.8%
Post‐deployment hedonic happiness			
Suffering	*B* = −0.112, *p* = 0.032	*B* = 0.003, *p* = 0.018	*B* = −0.086, *p* = 0.146
Zeal	*B* = 0.102, *p* = 0.106	*B* = −0.069, *p* = 0.060	*B* = 0.081, *p* = 0.278
Contentment	*B* = 0.202, *p* < 0.001	*B* = 0.057, *p* < 0.001	*B* = 0.192, *p* = 0.006
Restlessness	*B* = –0.146, *p* = 0.015	*B* = 0.053, *p* = 0.081	*B* = −0.161, *p* = 0.016
Explained variance	15.9%	16.1%	11.4%

*Note:* Each row presents results from robustness checks under three analytic conditions. In Column 1, covariates were excluded, but attention check filters and imputed data were retained. In Column 2, participants failing attention checks were removed, while covariates and imputed data were retained. In Column 3, raw (non‐imputed) data were used, with covariates and attention check filters included. *B* = unstandardised regression coefficient; *p* = significance level.

### 3.5 Summary of Findings

Overall, the findings support all three hypotheses. Perceptions of organizational affect align with a two‐dimensional structure of ambiance (emotional tone; valence) and vigour (motivational energy; activation), supporting H1, and four distinct perceptions of organizational affect emerged: zeal (positive ambiance, high vigour), contentment (positive ambiance, low vigour), restlessness (negative ambiance, high vigour), and suffering (negative ambiance, low vigour), supporting H2. These perceptions of organizational affect were differently related to indicators of holistic mental health, partially supporting H3.

## 4 Discussion

This study examined how perceptions of organizational affect align with the two‐dimensional models of social perception and core affect, and how these perceptions relate to holistic mental health across a military deployment cycle. Factor analyses confirmed a two‐dimensional structure of organizational affect, identifying four distinct perceptions—zeal, contentment, restlessness, and suffering—that emerge from the interplay between ambiance (emotional tone) and vigour (motivational energy). These findings support H1, demonstrating alignment with Social Perception Theory (Fiske et al. 2007) and Core Affect Theory (Russell 1980). The emergence of these four perceptions also supports H2, indicating that service members perceive organizational affect through distinct patterns that integrate emotional and motivational characteristics.

### 4.1 Nuanced Support for Hypothesis 3

Perceptions of organizational affect significantly predicted psychological distress, hedonic happiness, and eudaimonic thriving; however, these relationships were more complex than initially hypothesised. Perceptions of suffering (disgruntled, lethargic) were associated with increased psychological distress and decreased eudaimonic thriving, reinforcing its detrimental impact. However, suffering did not significantly predict hedonic happiness, suggesting that distress increased while general life satisfaction remained stable. Contentment (congenial, lethargic) was positively associated with hedonic happiness, but not with distress or thriving, suggesting a stable yet passive environment that fosters satisfaction but not growth. Perceptions of zeal (congenial, exuberant) and restlessness (disgruntled, exuberant) were associated with eudaimonic thriving, indicating that high‐energy environments, even those with emotional tension, can enhance engagement and psychological growth. Importantly, neither zeal nor restlessness was consistently associated with distress or happiness, suggesting that vigour plays a crucial role in fostering thriving regardless of emotional tone.

### 4.2 Interpretation of Findings

These findings suggest that different dimensions of organizational affect have distinct associations with different aspects of mental health and well‐being, and that motivational energy (vigour) crossed with emotional tone (ambiance) may be a more consistent predictor of thriving than either dimension alone. Perceptions of suffering were robustly associated with increased psychological distress and reduced eudaimonic thriving, but not with hedonic happiness, suggesting that such climates heighten internal distress without necessarily diminishing subjective life satisfaction. This may reflect military cultural norms that prioritise endurance and composure in the face of hardship. Notably, the stability of these associations across the mid‐ and post‐deployment phases suggests that the relationship between perceptions of organizational affect and holistic mental health holds across different operational contexts.

Perceptions of contentment, in contrast, predicted greater hedonic happiness but were not significantly associated with distress or thriving. This suggests that a low‐energy, emotionally positive climate may buffer stress and enhance day‐to‐day satisfaction without necessarily fostering growth or deeper meaning. Perceptions of zeal were positively associated with eudaimonic thriving, supporting the idea that energised, emotionally positive climates cultivate purpose and engagement. Interestingly, perceptions of restlessness also predicted eudaimonic thriving, suggesting that even climates marked by dissatisfaction or tension can promote growth, meaning, and purpose. However, zeal and restlessness did not predict distress or happiness, indicating that their energising effects are more narrowly linked to meaning and personal development. This pattern refines our understanding of how organizational affect functions: while ambiance shapes well‐being in intuitive ways (positive tone = happiness), it is vigour that drives personal growth, regardless of emotional valence.

### 4.3 Theoretical Contributions

This study offers a more granular understanding of organizational affect by showing that its components (suffering, contentment, zeal, and restlessness) have distinct and at times unexpected relationships with the different dimensions of holistic mental health. It advances Core Affect Theory (Russell 2003) by confirming that organizational climates are experienced along orthogonal dimensions of valence and energy. It also adds nuance to Social Perception Theory (Fiske et al. 2007) by demonstrating that judgements of organisations, like judgements of people, reflect combinations of warmth (ambiance) and competence (vigour), with different affective blends producing different outcomes. Crucially, the findings extend Challenge‐Hindrance Stressor Theory (LePine et al. 2005), suggesting that less‐than‐pleasant environments with high energy may operate as ‘challenge stressors’, contributing to thriving. This theoretical integration emphasises that energy, not just positivity, matters in fostering growth and resilience.

### 4.4 Practical Implications

This study focused on Soldiers assigned to a U.S. Army Armoured Brigade Combat Team (ABCT), a self‐contained unit comprising approximately 4000–4500 personnel. A brigade combat team is a ‘team of teams’ and is distinct in the Army hierarchy as it is the first echelon that incorporates differing functions into a single whole, including infantry, armour, artillery, engineer, and logistic battalions (U.S. Department of the Army 2021). Given its size, complexity, and operational demands, the ABCT functions as a distinct organizational environment shaped by shared stressors and a hierarchical command structure. Within this context, organizational affect likely reflects perceptions of the unit as a whole, with brigade‐ and battalion‐level dynamics shaping emotional tone and energy. As such, the practical recommendations below are especially relevant for brigade and battalion commanders, senior enlisted leaders, and supporting staff (e.g., operations, personnel, behavioural health). These findings may also apply to similarly structured civilian organisations, such as large hospitals, emergency response teams (e.g., law enforcement, fire, and emergency medical services), or regional logistics hubs.

These results offer differentiated guidance for leadership and organizational design. Rather than broadly promoting ‘positivity’, leaders should consider both the emotional ambiance and motivational vigour of the workplace. When perceptions of suffering dominate, leaders may benefit from focussing on restoring psychological safety (Edmondson and Lei 2014), rebuilding team cohesion (Kozlowski and Ilgen 2006), and reigniting motivation (Deci and Ryan 2000), as this affective climate is consistently associated with higher distress and lower thriving.

In contrast, when contentment prevails, leaders might introduce stretch goals or developmental opportunities to prevent stagnation (McCauley and Hezlett 2001; Ryan and Deci 2001). While this climate supports happiness, its low energy may limit growth and engagement (Csikszentmihalyi 1990).

Zeal, characterised by high energy and a positive emotional tone, was positively associated with eudaimonic thriving, suggesting that it may foster purpose, growth, and intrinsic engagement (Ryan and Deci 2000). Leaders can cultivate this affective climate by supporting autonomy, aligning teams around meaningful goals, and promoting energised collaboration (Deci et al. 2017; Edmondson and Lei 2014).

Interestingly, restlessness, marked by high energy and a negative tone, is not necessarily harmful. Leaders should recognise that dissatisfaction does not always signal dysfunction; when this restless energy is channelled productively through innovation sprints, problem‐solving initiatives, or leadership development programs, it may enhance psychological thriving (LePine et al. 2005).

Taken together, these results suggest that aligning interventions with specific affective profiles is more effective than applying one‐size‐fits‐all morale‐boosting strategies. Leaders should assess the emotional climate of their teams, identify areas where tone or energy is lacking, and tailor their approach accordingly.

### 4.5 Strengths and Limitations

This study offers valuable insights into the relationship between perceptions of organizational affect and holistic mental health in high‐stakes environments. The rigorous factor analytic approach confirmed the two‐dimensional structure of organizational ambiance and vigour, identifying four distinct perceptions: suffering, contentment, restlessness, and zeal. The study's multi‐time‐point design, use of validated mental health measures, and sensitivity analyses further enhance confidence in the robustness and generalisability of these findings within military contexts. Additionally, by integrating theoretical perspectives from social psychology, organizational behaviour, and occupational mental health, this research advances the conceptualisation of workplace affect, demonstrating how perceptions of emotional tone and motivational energy interact to shape holistic mental health.

Despite these strengths, several limitations must be acknowledged. The study's repeated cross‐sectional design, dictated by operational constraints, prevented the tracking of the same individuals over time, thereby limiting the ability to make causal inferences. Although our theoretical framework and analytic sequencing treat perceptions of organizational affect as predictors of mental health, this relationship may be bidirectional. Individuals experiencing higher distress may be more likely to perceive their organizational environment in a negative light. While our use of baseline affect measures to predict later outcomes supports temporal ordering, causal direction cannot be definitively established without within‐subject longitudinal data. Additionally, while the study identified four distinct perceptions of organizational affect, the nature of the data did not permit the use of latent profile analysis (LPA) or other clustering methods to confirm whether individuals' perceptions naturally coalesce around distinct profiles. Future research should employ longitudinal panel designs to assess how individual perceptions of organizational affect evolve and influence mental health outcomes, and explore whether changes in mental health predict shifts in perceived organizational affect, or vice versa. Person‐centred methods may also help identify meaningful subgroup and developmental trajectories across time.

The use of T1 data warrants additional explanation. T1 responses served two purposes: establishing the factor structure of the organizational affect measure and providing baseline covariates for analyses at later timepoints. However, some participants completed T1 but did not complete T2 or T3 and therefore contributed only to the exploratory factor analysis and covariate estimation in the general linear models. This approach leveraged the largest available sample for each analytic step but introduced complexity in interpreting how attrition may have influenced subsequent portions of the analysis. Although attrition between timepoints was expected given the operational tempo, it raises concerns about potential response bias. To evaluate this, we conducted an attrition analysis comparing participants who remained in the study with those who dropped out. Results showed no significant differences across demographic variables or baseline mental health indicators, with the exception that those who remained at T3 reported slightly lower perceptions of organizational suffering. This finding suggests that attrition was essentially random and unlikely to have meaningfully biased results.

The study also relied solely on self‐reported measures, introducing potential biases such as social desirability and common method variance (Krumpal 2013; Podsakoff et al. 2024). While anonymity and validated measures were used to mitigate these concerns, future research should integrate multi‐method assessments, such as peer evaluations, physiological indicators, or sentiment analysis from organizational communications, to further enhance the understanding of this phenomenon.

Additionally, the study focused on a U.S. Army ABCT, which may limit the generalisability of findings to other military units or civilian workplaces. Replicating this study across diverse occupational settings will help determine the broader applicability of the framework. Lastly, the composite measure of psychological distress (combining anxiety, depression, and PTSD symptoms) may have obscured unique relationships between specific distress types and organizational affect. Future research should disaggregate these constructs to explore how different forms of psychological distress relate to organizational ambiance and vigour.

Despite these limitations, this study provides a theoretically and empirically grounded framework for understanding how Soldiers' perceptions of workplace emotional climates influence holistic mental health. Refining methodological approaches, incorporating longitudinal designs, and broadening the scope of analysis will further illuminate how organizational affect shapes resilience, engagement, and performance in high‐stakes environments.

### 4.6 Future Directions

Building on these findings, future research should continue to investigate the causal mechanisms underlying the relationship between perceptions of organizational affect and holistic mental health. Longitudinal panel designs that track individual perceptions and outcomes over time would help clarify the stability and directionality of these relationships. Expanding this work across different military branches, operational units, and civilian organisations would enhance generalisability and reveal contextual differences in how perceptions of organizational climates influence holistic mental health.

Emerging analytic techniques such as machine learning and natural language processing offer promising avenues for capturing shifts in organizational affect. AI‐driven tools could analyse real‐time feedback, communication sentiment, and digital interactions to detect trends in collective affect and predict their impact on holistic mental health. While these methods may enhance early intervention capabilities, their ethical and practical implementation requires careful evaluation.

Individual differences such as personality traits, adaptive capacity, and prior stress exposure may moderate how perceptions of organizational affect influence holistic mental health. Future studies should examine whether Soldiers of different ranks, roles, or demographic backgrounds interpret and respond to organizational ambiance and vigour differently. Such insights could inform tailored interventions that account for diverse needs and experiences. Further theoretical development is also warranted. These findings support Affective Events Theory (Weiss and Cropanzano 1996) and Challenge‐Hindrance Stressor Theory (LePine et al. 2005). Future work should explore how perceptions of organizational affect evolve over time and distinguish between stable versus volatile patterns. Understanding these dynamics could reveal how emotional environments shape individual and team functioning in high‐stakes settings.

Finally, continued refinement and validation of assessment tools are essential. The novel measure introduced here captures two fundamental dimensions of perceptions of organizational ambiance and vigour, but should be tested across broader organizational contexts. Affective profile assessments can help leaders shape the workplace climate, align leadership practices with values and goals related to well‐being, and support strategic decision‐making.

## 5 Conclusion

This study presents a novel framework for understanding the relationship between perceptions of organizational affect and holistic mental health in high‐stakes environments. By conceptualising organizational affect along the two dimensions of ambiance (emotional tone) and vigour (motivational energy), we identified the four distinct perceptions of zeal (positive ambiance, high vigour), contentment (positive ambiance, low vigour), restlessness (negative ambiance, high vigour), and suffering (negative ambiance, low vigour). This framework provides a structured approach to understanding how individuals perceive their organisation's emotional climate and how these perceptions relate to holistic mental health.

These findings highlight the crucial role of vigour in fostering eudaimonic thriving. While perceptions of a positive ambiance support happiness and reduce distress, it is perceptions of high vigour, regardless of the ambiance's valence, that are most critical for growth and engagement. Conversely, perceptions of low‐vigour environments exhibit more variable effects, with contentment being linked to happiness, but not growth, and suffering associated with poorer outcomes related to distress and a lack of thriving. Importantly, these associations were consistent across both the operational and reintegration phases of deployment, underscoring the robustness of the findings.

These results refine theoretical models of affect and stress, extending insights from Social Perception, Core Affect, and Challenge‐Hindrance Stressor theories. Organisations, especially in high‐stakes contexts like the military, should assess and manage the perceptions of collective affect by cultivating high‐vigour, purpose‐driven environments. Future research should investigate how individual, contextual, and structural factors moderate these relationships and whether interventions that target ambiance and vigour can significantly enhance holistic mental health and performance over time.

## Author Contributions

W.J.S. designed the study, collected and analysed the data, and draughted the original manuscript. R.L.J. and S.P.T. contributed to the manuscript's writing, review, and editing.

## Ethics Statement

The Walter Reed Army Institute of Research Institutional Review Board (IRB) (Protocol #2460) approved this study.

## Conflicts of Interest

The authors declare no conflicts of interest.

## Data Availability

The Department of Defense policy prohibists sharing data and study materials associated with this research. In addition, consent documents state that only aggregate data will be shared outside the Walter Reed Army Institute of Research. However, data may become available from the corresponding author on reasonable request and with an approved data use agreement. We thank the Operational Research Team at the Walter Reed Army Institute of Research.
